# A Review on Inflammasomes and Immune Checkpoints in Pre-Eclampsia Complicated with Tuberculosis and Human Immune Deficiency Virus

**DOI:** 10.3390/ijerph20176627

**Published:** 2023-08-22

**Authors:** Wendy N. Phoswa, Olive P. Khaliq, Simeon Eche

**Affiliations:** 1 Department of Life and Consumer Sciences, Science Campus, University of South Africa (UNISA), Private Bag X 6, Florida, Roodepoort 1710, South Africa; 2 Department of Paediatrics and Child Health, University of the Free State, Bloemfontein 9300, South Africa; khaliqop@ufs.ac.za; 3 School of Medicine, Yale University, 333 Cedar Street, New Haven, CO 06510, USA; simeon.eche@yale.edu

**Keywords:** inflammasomes, immune check points, inflammation, tuberculosis, HIV, pre-eclampsia

## Abstract

The current review evaluates how inflammasomes and immune checkpoints are regulated in pre-eclampsia (PE) associated with tuberculosis (TB) and Human Immune Deficiency Virus (HIV). Studies indicate that inflammasomes such as (NRLP3, NEK7, and AIM2) and immune checkpoints such as (CLT4, PD-1, TIM3, and LAG-3) are dysregulated in TB- and HIV-infected individuals, and also in pre-eclamptic pregnancies, which explains why pregnant women who are either infected with TB or HIV have an increased risk of developing PE. Evidence suggests that inhibition of inflammasomes and immune checkpoints may assist in the development of novel anti-inflammatory drugs for the prevention and management of PE in patients with or without TB and HIV infection.

## 1. Introduction

Pre-eclampsia (PE) is one of the world’s leading maternal and fetal morbidity and mortality that affects 2–4% of all pregnancies [1]. This disorder is responsible for approximately 46,000 maternal deaths and 500,000 fetal deaths each year [1]. The clinical diagnosis of pre-eclampsia includes a blood pressure of (≥140/90 mmHg) accompanied by proteinuria of (≥300 mg/24 h) occurring at ≥20 weeks of gestation [2,3].

Pre-eclampsia has been classified as a two-stage disease model. Stage 1 is characterized by poor placental development as a result of inadequate trophoblast invasion with subsequent impaired spiral artery remodeling [4,5,6,7]. This then leads to a hypoxic or ischemic placenta, which is the primary cause of stage 2 of the disease also known as maternal systemic immune response [4,5,6]. Low oxygen levels in the hypoxic or ischemic placenta create an oxidative-stress-dominated environment, with abundant reactive oxygen species (ROS) in the placenta [8]. This alters the normal levels of angiogenic factors such as the placental growth factor (PGFI), vascular endothelial growth factor (VEGF), and angiopoietin, Which are responsible for normal placental development [9]. Reactive oxygen species also causes the maternal immune response to be exaggeratedly activated to maternal diseases, such as pre-eclampsia. It is quite clear that the placenta is the main organ that drives the pathogenesis of the disease, which explains why the only current effective treatment for PE is the delivery of the baby and the removal of the placenta [10].

Even though the actual etiology of pre-eclampsia remains unknown, several factors are known to be responsible for the development of pre-eclampsia. These are environmental, genetic, and immunological factors [11]. Current literature indicates that immunological markers are the most prominent contributors to the development of PE during pregnancy. These immunological markers can include immune checkpoints, inflammasomes, and cell-cycle markers. In pre-eclamptic pregnancies, these immunological markers can be influenced by several risk factors, such as pregnancy above the age of 35, smoking, obesity, African ancestry, pre-existing hypertension, previous history of pre-eclampsia, diabetes, primigravida, in vitro pregnancy, and other immune suppressive diseases like HIV infection and tuberculosis (TB) [11,12].

Evidence suggests that HIV infection can increase the risk of pre-eclampsia, possibly due to the chronic immune activation and inflammation that occur with HIV infection [11]. Additionally, some studies have suggested that the use of antiretroviral therapy (ART) during pregnancy may further increase the risk of pre-eclampsia [11,13], although other studies indicate that it is not clear whether ART or HIV is the main cause of pre-eclampsia [14].

Pre-eclampsia is a severe pregnancy complication characterized by high blood pressure and damage to organs, such as the kidneys and liver. It can be life-threatening for both the mother and baby if left untreated [15].

Tuberculosis is another condition widely associated with pregnancy complications such as pre-term labor, low birth weight, and pre-eclampsia. Similar to HIV-infected pregnant women, TB may be associated with an increased risk of developing pre-eclampsia due to the chronic inflammation and immune activation associated with TB infection. Additionally, TB infection can cause damage to the kidneys, which can contribute to the development of pre-eclampsia.

With increased research indicating an association between chronic inflammation with TB/HIV or TB–HIV co-infection in pre-eclamptic pregnancies, it is, therefore, necessary to investigate how immune markers are regulated in the presence of TB/HIV or both and link that with the risk of pre-eclampsia development.

The primary objective of this review is to explore the immunological markers present in pre-eclamptic pregnancies associated with TB and HIV, with a specific emphasis on immune checkpoints and inflammasomes. This comprehensive analysis aims to advance our understanding of the underlying pathophysiology of pre-eclampsia, both in women with and without TB/HIV infections. By enhancing our comprehension, we intend to refine diagnostic approaches for the condition and recommend urgent facilitation on the development of novel therapeutic interventions capable of ameliorating or preventing the ailment within the context of TB/HIV mono-infection or co-infection.

## 2. Inflammasomes and Immune Checkpoint Markers in TB/HIV-Associated Pre-Eclamptic Pregnancies

Gaining insights into the alterations within the immune system during pregnancy holds the potential to illuminate the intricacies of changes occurring at the interface between the maternal and fetal domains. These modifications are pivotal in sustaining both the pregnancy and the well-being of the mother throughout this period [16]. The shifts observed in the maternal immune system serve a dual purpose: safeguarding the mother while simultaneously mitigating any detrimental immune reactions aimed at the developing allogeneic fetus [16]. Orchestrating these adaptive immune changes are the components of the innate immune response, with inflammasomes standing out as a key player that shapes adaptive immunity [17]. Concurrently, immune checkpoint markers, acting within immunosuppressive signaling pathways, contribute to the maintenance of adaptive immunity and self-tolerance [18].

Given the essential roles of immune checkpoint markers and inflammasomes in preserving self-tolerance and regulating the magnitude of immune responses, this review aims to establish a connection between the immune system and adaptive immune responses in complex pregnancies involving HIV, TB, and hypertensive disorders. By exploring this interplay, we intend to provide a comprehensive understanding of the immune system’s involvement in intricate pregnancy scenarios.

## 3. Inflammasomes

Inflammasomes are multi-subunit complexes consisting of a sensor protein, an adaptor protein, and caspase-1 that assemble in the cytosol after sensing pathogen-associated molecular patterns (PAMPs) or damage-associated molecular patterns (DAMPs) [19,20,21]. The sensor proteins are divided into three structural domains: (1) The nucleotide-binding domain and leucine-rich repeat-containing proteins (NLRs); (2) The absence of melanoma 2 (AIM2)-like receptors (ALRs); (3) The pyrin receptor. The adaptor protein is an apoptosis-associated speck-like protein containing a caspase activation and recruitment domain (CARD), which serves as the connector protein to bring the sensor protein and caspase-1 together in the inflammasome complex [22,23,24]. The prominent inflammasomes that have been discovered so far include NLRP1, NLRP3, NLRC4, NLRP7, NLRP12, human NLRP2, NLRP7, IF116, murine NLRP6 and NLRP9b, AIM2, and PYD [24,25]. The most common inflammasome is NLRP3, due to being associated with a wide range of diseases [17,26]. The activation of the NLRP3 inflammasome is mediated by NEK7 [27].

Upon activation, the NLRP3 inflammasomes recruit and activate the protease caspase-1, which then cleaves and activates the pro-inflammatory cytokines interleukin-1β (IL-1β) and interleukin-18 (IL-18) as well as inducing pyroptosis, a type of programmed cell death [28]. Although inflammasomes are important in facilitating the process of pathogens removal from the host cell, exaggerated activation of inflammasomes is also a major problem. Overactivation of inflammasomes has been identified as the cause of many autoimmune diseases, neurodegenerative disorders, and metabolic disorders such as cardiovascular diseases, type 2 diabetes, and obesity [21,29]. Therefore, inflammasomes have emerged as attractive targets for developing new therapeutics.

### 3.1. Inflammasomes and Pregnancy Development

Recently, the activation of inflammasomes has been linked with increased risk of pregnancy complications such as pregnancy-induced hypertension or pre-eclampsia. Under normal pregnancy conditions, inflammasomes play a role in pregnancy development by balancing the state of inflammatory response [30]. The role of inflammatory response in early stages of pregnancy development is to ensure adequate cytotrophoblast invasion in order to maintain a healthy perfusion in the placenta [31]. This also allows the mother to be tolerant to fetal development and to protect the mother from some other infectious diseases [32]. Under abnormal conditions, where there is extreme elevation of inflammasomes, the inflammatory response is also exaggerated, resulting in pregnancy-related complications such as pre-eclampsia [33,34].

Pre-eclampsia is an inflammatory condition leading to high blood pressure during pregnancy. The blood pressure in PE is usually greater or equal to 140 mmHg and proteinuria [1]. Although there are many factors that can lead to the development of pre-eclampsia, immunological factors have been of great interest, especially since other immunological conditions, such as HIV infection and TB have been associated with the development of PE. All evidence linking PE with HIV or TB points towards disturbances in the immune system. Even though other new mechanisms are still being discovered, it is important to know exactly why women infected with TB, HIV, or both have a high risk of PE development. With growing interest in inflammasomes, this review will explore how inflammasomes play a role in PE development in pregnant women affected by these diseases.

### 3.2. Inflammasomes in Pre-Eclampsia

Pregnancy involves a series of events, including the activation of the immune response to accommodate the growing fetus. However, in some cases, the immune response becomes overly activated, which poses serious health consequences to both the mother and the fetus. Pre-eclampsia, one of the health-threatening conditions in pregnancy, is suggested to occur due to disturbances in the immunological factors or markers. Inflammasomes have been identified as one of the immunological markers that are involved in the pathophysiology of PE. A study by Weel et al. [33] indicated that there was an increase in NLRP3 inflammasomes in women with severe pre-eclampsia. It has also been reported to contribute to the mechanism of placental inflammation in pre-eclampsia [35]. Moreover, gene expression of NRLP3 receptors was found to be increased in monocytes from pre-eclamptic women [36].

Increased inflammation in pregnancy can also be attributed to diseases such as tuberculosis and HIV infection. Both TB and HIV have been reported to be associated with poor pregnancy outcomes such as PE [37,38]. Inflammasomes have also been implicated in the pathogenesis of TB and HIV, which explains why pregnant women with one of these conditions are at risk of developing PE [37,38].

Within this present review, our focus will be on investigating the control mechanisms governing inflammasomes in the context of TB mono-infection, HIV infection, or the co-infection of TB and HIV. This endeavor aims to enhance our comprehension of the underlying factors contributing to the heightened vulnerability of women afflicted by either one or both of these conditions to pre-eclampsia.

### 3.3. Inflammasomes in Tuberculosis

Inflammasomes are activated upon exposure to a broad range of toxins, such as nigericin [39], fungi [40], bacteria [41], and viruses [42]. Firstly, the toxin enters the host cell. This stimulates DAMPs and PAMPs receptors which are found on the macrophages and neutrophils. Activation of these receptors results in the release of pro-inflammatory cytokines, reactive oxygen species, and nitrogen radicals, which play a significant role in pathogen removal, cell proliferation and immune response from the host cells.

In tuberculosis, inflammasomes play an integral role in fighting the mycobacterium infection [43,44,45,46]. Activation of inflammasomes triggers the release of pro-inflammatory cytokines in order to fight against bacterial infection. Over activation of inflammasomes has been observed in the pathology of TB and TB immune reconstitution syndrome [47]. Macrophages infected with mycobacterium tuberculosis activate the NLRP3 and AIM2 inflammasomes, which leads to an increased in IL-1β and IL-8 [48,49,50,51,52]. A study conducted on active TB patients indicated that IL-1 β and TNF-α were increased in the bronchoalveolar cells and this led to inflammation of alveolar cells [53].

Currently, to the best of our knowledge, there are no studies that have reported on inflammasomes in TB-associated pre-eclamptic pregnancies. However, one study indicated that latent TB-positive pregnant women had increased systemic inflammation, which was indicated by elevated levels of IL-1β when compared to latent TB-negative women [54]. Increased systemic inflammation is known to be associated with the pathogenesis of pre-eclampsia [55,56]. Giving attention to the inflammasome pathway in pregnancies complicated with TB is crucial as research has shown that the recognition of infectious agents such as in MTB by inflammasomes may lead to the initiation of a potent immune response [57,58], thus adding to the already exaggerated maternal immune response with negative consequences for the developing fetus.

### 3.4. Inflammasomes in HIV

Studies have shown that HIV infection can impair inflammasome activation [59,60]. For example, it was found that NLRP3 plays an essential role in CD4^+^ T cell loss in HIV-1–infected patients [61] and that HIV infection acts as a stimulator for NLRP3 and leads to cardiovascular-related complications [59]. Additionally, the AIM2 inflammasome was associated with increased production of pro-inflammatory cytokines, such as IL-1β and IL-18, in HIV patients [62]. Interestingly, dysregulation of inflammasome activation has been linked to the development of immune reconstitution inflammatory syndrome (IRIS), a paradoxical inflammatory reaction that can occur in HIV-infected individuals starting antiretroviral therapy (ART) and receiving treatment for TB [63,64].

The pathogenesis of IRIS is not completely understood, but it is thought to involve the dysregulation of the immune response to residual pathogen antigens. During HIV infection, the immune system is severely depleted, and the restoration of immune function during ART can result in an exaggerated inflammatory response to residual pathogen antigens. This can lead to the release of pro-inflammatory cytokines, such as interleukin-6 (IL-6) and tumor necrosis factor-alpha (TNF-α).

**Figure 1:**
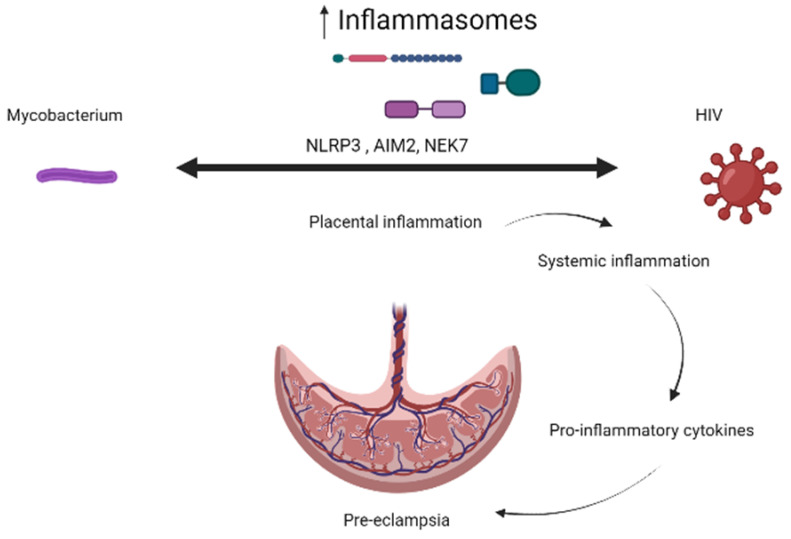
The schematic diagram indicates the role of inflammasomes in the pathogenesis of pre-eclampsia in the presence of tuberculosis or HIV infection. Mycobacterium mono-infection, solitary HIV infection, or the co-infection of TB and HIV have the potential to trigger prolonged activation of placental inflammasomes such as NLRP3, AIM2, and NEK7. Consequently, this sustained activation can induce an intensified inflammatory response within the placenta. This local inflammation can subsequently extend to cause systemic inflammation in the mother, marked by elevated levels of pro-inflammatory cytokines. The heightened peripheral pro-inflammatory cytokine levels can ultimately contribute to the onset of pre-eclampsia.

Increased activation of pro-inflammatory cytokines has been previously linked with adverse maternal effects such as PE. Therefore, one of the reasons why HIV-infected women have higher chances of developing PE is likely to be caused by IRIS (Figure 1).

### 3.5. Inflammasomes in TB–HIV Co-Infection

Although there is little information on the role of inflammasomes in TB–HIV co-infected, a study from Oxford University observed that an inflammasome NLRP3 gene polymorphism (rs10754558-G SNP) is associated with an increased risk for early mortality in HIV–TB co-infected patients initiating ART [65]. Similarly, this can be attributed to an increased maternal and fetal mortality rate in pregnant women infected with TB–HIV co-infection. Research findings show that pregnant women who are living with HIV and concurrent TB infection have an increased risk of developing pregnancy complications such as pre-eclampsia and pre-term delivery, amongst others [66].

## 4. Inflammasomes in the Presence of HIV and Tuberculosis Treatment

In individuals living with HIV infection undergoing highly active antiretroviral treatment (HAART), there has been documented upregulation of inflammasomes. In a study by Bandera et al. [67], an increase in NLRP3 and caspase-1 levels was observed specifically in immunological non-responder (INR) patients. These INR patients are defined as those on HAART with compromised immune reconstitution due to low circulating CD4^+^ T cell counts despite viral load suppression.

A similar investigation comparing NLRP3 inflammasome levels between HAART-naïve individuals and healthy controls revealed analogous findings. After treating HAART-naïve patients for 6 months with elvitegravir/cobicistat/emtricitabine/tenofovir disoproxil fumarate, a reduction in caspase-1, a marker of NLRP3 activation, was noted. Intriguingly, even post HAART treatment, these patients still displayed heightened NLRP3 inflammasome activation compared to healthy controls [68]. This suggests that inflammasome activation in HIV-infected individuals is potentially driven more by the virus itself than by treatment.

Supporting this notion, Guerville et al. [69] demonstrated elevated levels of inflammasome activation markers such as IL-1β and IL-18 in the serum of patients with long-term effective ART, contrasting with healthy controls.

Despite the existing knowledge about inflammasome regulation in the context of HIV infection and HAART treatment, there remains a dearth of evidence concerning the regulation of inflammasomes during TB treatment.

## 5. Immune Checkpoints and Their Role in Pregnancy

Immune checkpoints are immunosuppressants that prevent inflammation. When activated, the checkpoints inhibit pro-inflammatory cytokine release [70]. Other physiological functions include the prevention of autoimmunity and tumors from attacking the immune system [70]. During pregnancy, the maternal immune response is activated to protect the body against infections and to prevent fetal rejection by the mother’s antigens. The fetus carries maternal and paternal DNA material; therefore, the mother’s body does not recognize the DNA material from the paternal side and regards the fetus as a foreign body [71]. Immune checkpoints are activated as early as implantation to prevent an attack from the maternal antigens. The four commonly studied are the cytotoxic T-lymphocyte–associated antigen 4 (CTLA-4), the programmed death 1 (PD-1) receptor, and the T cell immunoglobulin and mucin-domain containing-3 (TIM-3) and the lymphocyte activation gene 3 (Lag-3) [71].

### 5.1. Cytotoxic T-Lymphocyte-Associated Antigen 4 (CTLA-4)

The immune checkpoint activated during implantation is the cytotoxic T-lymphocyte-associated antigen 4 (CTLA-4). The CTLA-4 is located on the surface of the T-regulatory cells (Tregs). T-regulatory cells control inflammation. Since pregnancy activates an immune response, Tregs are elevated in early pregnancy, highlighting their important role in pregnancy. A study reported that limited Tregs or diminished Tregs lead to implantation failure, fetal growth restriction, or fetal death [71]

The elevated Tregs in early pregnancy result in a subsequent increase in CTLA-4 cells in the peripheral area of Treg cells [72,73] These cells bind to their B-ligands (CD80/86) and initiate the expression of essential cells such as the Indoleamine 2, 3-dioxygenase (IDO), for early pregnancy development [73,74]. The IDO enzyme stimulates maternal tolerance at the maternal-fetal interface [75].

### 5.2. The Programmed Death 1 (PD-1) Receptor

The programmed death 1 (PD-1) receptor is another immune checkpoint located on the surface of various immune cells [76,77]. These cells promote Treg cell differentiation and enhance immune response repression [77]. Since this is a receptor, it requires binding to a ligand to be active. There are two PD-1 ligands, programmed death ligand 1(PD-L1) and programmed death ligand 2 (PD-L2). Programmed death ligand 1 is found on the placenta, specifically, the syncytiotrophoblast cells and villous cytotrophoblast cells, while PD-L2 is predominantly found in the villous cytotrophoblast cells [78,79]. The PD-L1 is elevated throughout pregnancy, with a higher expression noted during 12 weeks of gestation [80].When PD-1 binds to PD-L1, pro-inflammatory cytokine production in the CD4^+^ T cells is decreased, inhibiting immune response in the maternal-fetal interface [71].

### 5.3. T Cell Immunoglobulin and Mucin-Domain Containing-3 (TIM-3)

T cell immunoglobulin and mucin-domain containing-3 (TIM-3), an immune checkpoint found on surfaces of immune cells like the natural killer cells (NK cells), Type-1 T helper cells (Th1), Type-17 T helper cells (Th17), natural killer T-like cells (NKT-like cells), Tregs, and antigen presenting cells (APCs) [81]. The TIM-3 immune checkpoint plays a role in both activation and inhibition of the immune response [82,83]. TIM-3 inhibits Th1 activation in pregnant women to hinder a pro-inflammatory response [84]. For TIM-3 to function, it requires binding to its ligand, the galetin-9 (gal-9), to form a TIM-3/gal-9 pathway. This pathway promotes Th-1 and Th-17 cell death for immune tolerance and is reported to create a balance between Th1/Th2 [85].

### 5.4. Lymphocyte Activation Gene 3 (Lag-3)

Lymphocyte activation gene 3 (Lag-3) is an immune checkpoint identified to resemble CD4^+^ cells in structure [86]. This gene binds to two ligands: the major histocompatibility complex II (MHC II) and the fibrinogen-like protein 1 (FGL-1). The Lag-3 receptor prevents T cell proliferation and activation when bound to either one of the ligands [87,88]. Lymphocyte activation gene 3 is also expressed in Treg cells. In pregnancy, the Lag^3+^Treg cells are found in the periphery and the decidua during the early stages of pregnancy and are reported to inhibit proliferation from stimulating maternal tolerance against the fetus [71].

## 6. Immune Response in Pre-Eclampsia

In normal pregnancies, the immune cells are regulated toward maternal tolerance to the fetus with the aim to create a safe environment [32]. Maternal tolerance promotes uterine spiral artery remodeling, resulting in increased uteroplacental blood flow for proper fetal development [32]. However, in the case of pre-eclampsia, the contrary occurs. The immune cells are activated to prevent maternal interlace leading to a high inflammatory state that affect vasculature. Uterine spiral artery remodeling and trophoblast invasion are interrupted, leading to placental ischemia, oxidative stress, hypoxia, intrauterine growth restriction and maternal hypertension [89]. Immune checkpoints are reported as pro-inflammatory cytokine inhibitors, their role in a pre-eclamptic environment is of interest.

### 6.1. Cytotoxic T-Lymphocyte-Associated Antigen 4 (CTLA-4) in Pre-Eclampsia

The CTLA-4 receptor is located on the surface of Treg cells. In PE, Treg cell expression is lower in the periphery and decidua than normal pregnancies. Furthermore, their immunosuppressive effect is impaired, leading to increased inflammation, a known feature of PE [90,91]. Surprisingly, there was no difference in the expression level of CTLA-4 on Treg cells in PE compared to normotensives. However, CD 80 and CD86, CTLA-4 ligands, have been noted to increase in a PE [70]. Therefore, the role of CTLA-4 in PE is poorly understood and yet to be elucidated [71].

A study by Jaaskelainen et al. [92] explored the association of CTLA-4 gene polymorphism (49A-G polymorphism (dbSNP: rs231775)) with the development of PE. The results showed that the G allele had a higher frequency in PE compared to normal pregnancies and a significant decrease in AA genotype was associated with placental abruption. Furthermore, another study that investigated the same polymorphism in severe PE reported a significant increase in the frequency of the AG heterozygous genotype in severe PE compared to normotensives [93].

### 6.2. The Programmed Death 1 (PD-1) Receptor in Pre-Eclampsia

The role of PD-1 in PE needs to be clarified. However, PD-1 is upregulated in Treg cells of women with PE even though Treg cells are less in PE [94]. A study reported that the ligand PDL1 expression remained the same in both PE and normotensives [95]. In Th17 cells that induce inflammation during microbial invasion, PD-1 is upregulated but remains the same in other cells, such as CD4^+^ and CD3^+^. It was concluded that the PD-1/Th17 pathway was impaired in women with PE [91,96]. In addition, an upregulation of PD-1 expression was reported in Treg cells of PE women compared to normotensives [97,98]. Soluble PD-1(sPD-1) expression was higher in PE compared to normotensives. In women with <34 weeks of gestation, sPD-1 was significantly increased in PE compared to normotensives, while women in >34 weeks showed a higher level of sPD-1 with no statistical significance [99,100]. Cells identified with high PD-1 expression in early pregnancy were CD4^+^, CD8^+^, Tregs, and NKT-like cells, suggestive of its inability to prevent Th1 reactions and promote immune response activation in PE [101].

### 6.3. T Cell Immunoglobulin and Mucin-Domain Containing-3 in Pre-Eclampsia

The expression of TIM-3 in early-onset PE was explored, and it was discovered that TIM-3 was significantly downregulated in NK cells, T cells, cytotoxic T cells, and CD53dim compared to normotensive pregnancies [102]. Furthermore, NK cells and cytotoxic T cells expressed higher cytotoxicity than in normal pregnancies [102]. The TIM-3/Gal-9 pathway is dysregulated in PE and contributes to the exaggerated immune response noted in PE [102].

During pregnancy, the decidual immune cells express lower levels of TIM-3 cells in PE than in normotensives [103]. Interestingly, normotensive pregnancies have a higher expression of anti-inflammatory cells and a decreased expression level of pro-inflammatory cytokines [103]. Tests revealed that TIM-3 hinders pro-inflammatory cytokine production since its blockade increases pro-inflammatory cytokine production and decreases anti-inflammatory cells [103]. T cell immunoglobulin and mucin-domain containing-3 promotes tubal formation; when inhibited, it affects this process, indicative of the insufficient spiral artery remodeling noted in PE [104].

### 6.4. Lymphocyte Activation Gene 3 (Lag-3) in Pre-Eclampsia

Lymphocyte activation gene 3 promotes immune tolerance in pregnancy. However, in pre-eclampsia its role requires investigation. Liang et al. [105] reported a non-significant difference in CD3^+^ T cells in normotensive pregnancy compared to PE complicated pregnancies. Moreover, CD4^+^ and CD8^+^ cells showed a low expression of Lag-3 in early-onset PE compared to normotensive pregnancies [105]. The Lag-3/FG-L pathway plays a role in the pathogenesis of the PE [105]. Other findings report the upregulation of FG-L in pregnancy decreases PE progression by inhibiting T cell functioning to decrease the production of pro-inflammatory cytokines and promote immunotolerance [106]. Circulatory FG-L is upregulated in early-onset PE to counteract the progression of the disease by promoting hepatocyte and trophoblast multiplication [104].

Pregnant women are at higher risk of other bacterial infections such HIV and tuberculosis (TB). Both these infections trigger an immune response. Tuberculosis can either be active or latent (infected but not actively ill) [107]. Active TB is found in only 5–10% of the infected population, while 90–95% of the infections remain latent [107]. The transition from latent to active TB is influenced by immunological factors such as immune checkpoints during infection [108]. During TB infection, CD4^+^ and CD8^+^ cells are activated to produce interferon-gamma to produce cytokines to fight off infection [109] Latent TB can also be transformed into active TB by the human immune deficiency virus (HIV). Viral invasion into the host cell decreases immunological function, thus activating latent TB. Patients with HIV are 20–40 times more likely to have active TB than those without HIV [110].

Pregnancy has also been reported to trigger the reactivation of latent TB into active TB [111]. The immune system is suppressed for fetal survival during pregnancy, allowing pathogen invasion in the maternal system. This may be transmitted to the fetus through the placenta. It has been reported that maternal TB can increase neonatal TB infection. Furthermore, pregnant women with HIV are even at a higher risk of TB infection than those without HIV [112].

Immune checkpoint inhibitors are activated during a viral or bacterial infection. During TB infection, T lymphocytes activate macrophages to kill the bacteria [113]. If killing of the bacteria is unsuccessful, a granuloma is formed, leading to latent TB. However, this process activates immune checkpoint inhibitors. Wang et al. [114] reported elevated PD1, TIM3, and CTLA-4 expression levels on CD4^+^ and CD8^+^ cells in latent TB compared to controls. The treatment of latent TB decreases immune checkpoints, CD4^+^, CD8^+^, lymphocytes, and macrophages [114]. Interestingly, immune checkpoint inhibitors therapy has been reported to result in active TB in individuals with latent TB [115].

Whilst elevated immune checkpoints inhibitors prevent active TB, the contrary occurs in HIV infection. During HIV infection, T cells, specifically CD8^+^ T cells, are activated to fight off infection. In early stages of infection, these cells are able to inhibit viral replication. However, the more the viral antigens the more difficult it becomes for the T cells to suppress replication [116,117]. In HIV-positive individuals, immune checkpoints are elevated on CD8 T cells [118]. Chronic HIV infections are reported to positively correlate with PD-1, which increases with increasing viral load and inversely correlates with CD4 count. The PD-1 immune checkpoint inhibitor may be used as an indicator of CD8 T cell impairment [119]. The PD-1 pathway is fully functional during active HIV infection to prevent an immune response. When the pathway is blocked CD8 T cells and CD4 T cells are activated to elicit an immune response [120].

Other immune checkpoint inhibitors such as the CTLA-4 cells are also elevated on CD4 T cells in HIV positive individuals than in HIV negative individual [117]. Like PD-1, blockage of CTLA-4 restores CD4 T cell function [121].The T cell immunoglobulin and mucin domain-containing protein 3 (TIM-3) also plays in role in suppressing CD4 T cell function in HIV-positive individuals. The expression levels of TIM-3 have been reported to rise with viral load and decrease with increasing CD4 T cell count [117]. Blocking the TIM-3 immune checkpoint also results in the activation of T cells to produce cytokines [122].The LAG-3 immune checkpoint is also elevated in HIV infection to suppress CD4 and CD8 T cell function. However, the use of combination antiretroviral therapy (cART) for long periods of time decreases the expression levels of LAG-3 while blockage of LAG-3 increases CD4 and CD8 expression (Table 1).

## 7. Immune Checkpoint Inhibitors in Pregnancy

The role of immune checkpoints in pregnancy is vital since it protects the fetus against an attack from the maternal immune response [123]. However, controversies exist regarding the blockage of these immune checkpoints since this would result in a maternal response against the fetus [124]. Furthermore, other studies have proved that blockage of PD-1/PD-L1 in monkeys during pregnancy dysregulates the maternal immune tolerance towards the fetus, resulting in death [125,126,127]. Also, immune checkpoint inhibitors have been suggested to cross the placental barrier, increasing the risk of fetal disorders [123]. This is an indication that immune checkpoint inhibitors are not recommended during pregnancy [128]. However, the use of these inhibitors has been reported as beneficial if used before pregnancy, or after conception as most babies are born healthy with no fetal abnormalities [129,130,131]. On the contrary, other data published found that the use of immune checkpoint inhibitors resulted in premature deliveries, fetal distress syndrome, spontaneous abortions, and intrauterine growth restriction [131].

The expression levels of the immune checkpoints are dysregulated during infection. Patients with PE have shown a significant decrease in CTLA-4 an increase in PD-1 in Treg cells, a decrease in TIM-3, and a decrease in LAG 3. In TB and HIV infections, all immune checkpoints were over expressed [94,102,103,104,117].

## 8. Future Recommendations

Inflammasomes and immune checkpoints regulate the immune response by increasing inflammation during infection. In pregnancy women, the level of inflammation may lead to PE. Future studies that involve therapeutic interventions that target inflammasomes and immune checkpoints would be beneficial to regulate the immune response during pathogen invasion.

## 9. Conclusions

Innate immune response components, namely inflammasomes and immune checkpoint markers, hold pivotal roles in the progression of pregnancy. Understanding the shifts in immunological dynamics during pregnancy becomes paramount for unravelling the intricacies at the maternal-fetal junction. These shifts contribute significantly to the maintenance of both pregnancy and maternal well-being throughout this period.

Moreover, within contexts characterized by heightened immune responses, such as in cases of HIV, TB, mono-, or co-infections, the presence of inflammasomes and immune checkpoint markers could potentially give rise to complications in pregnancy, like pre-eclampsia. Despite the potential viral load suppression achieved through HIV treatment, it is important to highlight that HAART therapy might not effectively mitigate the activation of inflammasomes. Consequently, women infected with HIV might experience exacerbated inflammation, underscoring the complexities of their condition.

## Figures and Tables

**Table 1:** Summary of immune checkpoints expression in PE, TB, and HIV.

Immune Checkpoints	PE	TB	HIV
CTLA-4	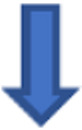	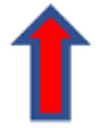	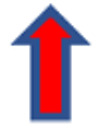
PD-1	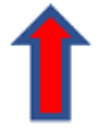	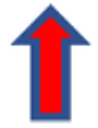	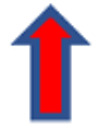
TIM-3	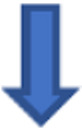	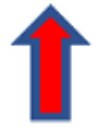	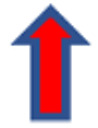
LAG-3	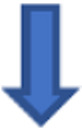	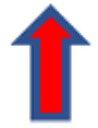	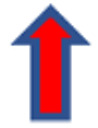

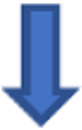
 = decrease and 
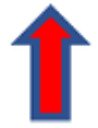
 = increase.

## Data Availability

Data sharing is not applicable to this article as no datasets were generated or analyzed during the current study.
